# Supplementary material for: A robust intrusion detection system based on a shallow learning model and feature extraction techniques
Source: PLoS One. 2024 Jan 24;19(1):e0295801. doi: 10.1371/journal.pone.0295801 (PMC10807775; doi:10.1371/journal.pone.0295801)
Supplement: Attachment — Submitted filename: Response to Reviewers.docx [file pone.0295801.s001.docx]

Rabat, November 15^th^, 2023

Mujeeb Ur Rehman, Ph.D.
Academic Editor
PLOS ONE

**COVER LETTER**

Dear Dr Mujeeb,

Enclosed, please find our revised article entitled “Enhancing Intrusion Detection Using Shallow Learning and Feature Extraction”. We are grateful to your consideration of our manuscript and the suggestions made by the reviewers.

We found that the comments were fair, carefully considered, and have helped to improve the work. We have now made substantial changes to the work, including additional data and improving the discussion and conclusion.

We have addressed all reviewer comments point-by-point. We thus feel the manuscript is greatly improved and are hopeful that it is now suitable for publication in PLOS ONE Journal.

Thank you for your consideration.

Best regards

**Samira Douzi, PhD**

**E-mail :** [s.douzi@um5r.ac.ma](about:blank)

**Responses to reviewer comments point by point**

**Reviewer 1**

CONSIDERATIONS

1) The abstract section is fragile. Please re-write an abstract section, explain an obtained result and contribution, improve a proposed method, etc. Please delete unnecessary information

**Thank you for your comment. The abstract has been modified, taking your feedback into consideration.**

2) This paper has more than spelling and grammatical errors. Please fix all of them.

**We have checked and corrected the grammatical errors.**

3) The authors should clearly state the limitations of the proposed method in other real applications.

**Thank you for your pertinent comment, please refer to paragraph 4.3.4.**

4) I suggest the authors add a table at the end of the literature review and compare the reviewed papers to clarify the research gap better.

**Thank you for this pertinent comment, which has been added to the Related Work section.**

5) The capitalization format of the title needs to be standardized. You can either capitalize the first letter of every word or use all lowercase letters.

**For all titles, we proceeded to capitalize the first letter of every word.**

6) Please re-write your contribution to this paper in the Introduction section.

**Thanks for the relevant comment, please see the introduction section.**

7) Please draw a flowchart of the proposed method.

**Thank you, the architecture of our approach already exists; refer to Figure 3.**

8) How did the authors set parameters for their proposed algorithm? Please make sensitivities of these parameters to the performance of their proposed algorithm!

**Thank you for these interesting comment, the parameters of our proposed algorithm were chosen based on our own experience**

9) Please make the Introduction and related work sections more productive using the following articles. Reading and using these articles and also cited in this article: A Multi-Objective Mutation-based Dynamic Harris Hawks Optimization for Botnet Detection in IoT, MOAEOSCA: an enhanced multi-objective hybrid artificial ecosystem-based optimization with sine cosine algorithm for feature selection in botnet detection in IoT, A Feature Selection based on the Farmland Fertility Algorithm for Improved Intrusion Detection Systems

**Thank you for this pertinent comment, which has been added to the Related Work and references section.**

10) Expand the critical results in the conclusion. Focus on the main developments in the finale. Also, write the main contributions in the conclusion.

**Thanks for the relevant comment, please see the Conclusion and Future Works section**

11) Numerical results are good enough, but more explanations are required to analyze each figure presented.

**Thanks for your comment, please see the Experimental Results and Discussion section**

12) The simulation section is not detailed enough. Authors are suggested to provide more information about their employed data and simulation process.

**Thank you for these interesting remarks, please see the Experimental Results and Discussion section.**

13) Please change the title of the end section (Conclusions) to (Conclusions and Future Works) and write some future works.

**Thank you for this pertinent comment, please see Conclusion and Future Works section.**

14) All figures have low quality, and please improve all of them.

**Thank you for your comment, the quality of the figures has been improved.**

**Reviewer 2**

CONSIDERATIONS

1) Why is shallow learning combined with feature extraction used to solve detection problems, rather than end-to-end deep learning? The author should explain this issue in depth from both theoretical and practical perspectives.

**Thank you for your comment. Please refer to the end of the "Materials and Methods" section.**

2) The specific structure of machine learning models should be introduced in detail. The reasons for adopting specific model structures and feature combinations should be explained.

**Thank you for your comment, kindly see paragraph 4.3.2.**

3) Many figures are blurry and should be re displayed.

**Thank you for your comment, the quality of the figures has been improved.**

4) Some deep learning methods should be considered for comparison.

**Thank you for this pertinent comment; we compared our proposed model with LSTM because, according to the state of the art and based on our knowledge, it is widely used in the field of intrusion detection. We also conducted comparisons with previous studies that utilized other types of deep learning algorithms, such as DNN, RNN, and CNN.**

5) Since deep learning methods have achieved remarkable results in the field of feature extraction and learning. The following related work of deep learning techniques must be cited and discussed, including “Spectrum interference-based two-level data augmentation method in deep learning for automatic modulation classification,” Neural Computing & Applications, vol. 33, pp. 7723-7745, 2020. “MR-DCAE: Manifold regularization-based deep convolutional autoencoder for unauthorized broadcasting identification,” International Journal of Intelligent Systems, vol. 36, no. 12, pp. 7204-7238, 2021. “Fine-grained modulation classification using multi-scale radio transformer with dual-channel representation,” IEEE Communications Letters, vol. 26, no. 6, pp. 1298-1302, June. 2022. Application of wavelet-packet transform driven deep learning method in PM2. 5 concentration prediction: A case study of Qingdao, China. Sustainable Cities and Society, 2023, 92: 104486. “DL-PR: Generalized automatic modulation classification method based on deep learning with priori regularization,” Engineering Applications of Artificial Intelligence, 2023, 122: 106082.

**Thank you for these interesting remarks, please see the introduction, where references have been added.**
